# Verifying likelihoods for low template DNA profiles using multiple replicates

**DOI:** 10.1016/j.fsigen.2014.06.018

**Published:** 2014-11

**Authors:** Christopher D. Steele, Matthew Greenhalgh, David J. Balding

**Affiliations:** a UCL Genetics Institute, Darwin Building, Gower Street, London WC1E 6BT, UK; b Orchid Cellmark Ltd., Abingdon Business Park, Blacklands Way, Abingdon OX14 1YX, UK

**Keywords:** Low-template DNA, DNA mixtures, Likelihood ratio, Replicates, Forensic, likeLTD

## Abstract

To date there is no generally accepted method to test the validity of algorithms used to compute likelihood ratios (LR) evaluating forensic DNA profiles from low-template and/or degraded samples. An upper bound on the LR is provided by the inverse of the match probability, which is the usual measure of weight of evidence for standard DNA profiles not subject to the stochastic effects that are the hallmark of low-template profiles. However, even for low-template profiles the LR in favour of a true prosecution hypothesis should approach this bound as the number of profiling replicates increases, provided that the queried contributor is the major contributor. Moreover, for sufficiently many replicates the standard LR for mixtures is often surpassed by the low-template LR. It follows that multiple LTDNA replicates can provide stronger evidence for a contributor to a mixture than a standard analysis of a good-quality profile. Here, we examine the performance of the likeLTD software for up to eight replicate profiling runs. We consider simulated and laboratory-generated replicates as well as resampling replicates from a real crime case. We show that LRs generated by likeLTD usually do exceed the mixture LR given sufficient replicates, are bounded above by the inverse match probability and do approach this bound closely when this is expected. We also show good performance of likeLTD even when a large majority of alleles are designated as uncertain, and suggest that there can be advantages to using different profiling sensitivities for different replicates. Overall, our results support both the validity of the underlying mathematical model and its correct implementation in the likeLTD software.

## Highlights

• The behaviour of multi-replicate LRs with respect to the inverse match probability is proposed as a method to validate forensic LR software.
• We perform lab-based and simulated experiments of one-, two- and three-contributor CSPs, as well as investigating a real-world CSP.
• LRs rise towards the IMP with additional replicates, while never exceeding it. Additionally, the LR from multiple low-template replicates can exceed that from a single good-quality sample.
• We validate likeLTD by demonstrating that it adheres to the expected behaviours.

## 1 Introduction

In forensic DNA profiling, a likelihood ratio (LR) is calculated to measure the support provided by DNA evidence (*E*) for a proposition *H*_*p*_ favouring the prosecution case, relative to its support for *H*_*d*_ representing the defence case. The LR can be written as

(1) $$\mathrm{LR}=\frac{\mathrm{Pr}(E|H_{p})}{\mathrm{Pr}(E|H_{d})}.$$

Each of *H*_*p*_ and *H*_*d*_ specifies a number of unprofiled contributors and a list of contributors whose DNA profiles are known (included in *E*). Typically *H*_*p*_ includes a profiled, queried contributor that we designate Q, who is replaced under *H*_*d*_ by an unprofiled individual X. Q may be an alleged offender, or a victim, while X is an alternative, usually unknown, possible source of the DNA. It usually suffices to limit attention to *H*_*p*_ and *H*_*d*_ that differ only in replacing Q with X, otherwise the LR is difficult to interpret as a measure of the weight of evidence for Q to be a contributor of DNA.

In addition to reference profile(s), of Q and possibly other known contributors, the DNA evidence consists of one or more profiling runs performed on a DNA sample recovered from a crime scene, or from an item thought to have been present when the crime occurred. Each profiling run generates graphical results in an electropherogram (epg), which we assume has been interpreted by a forensic scientist who decides a list of alleles observed at each locus, and also a list of potential alleles about which there is substantial uncertainty, perhaps due to possible stutter. Alleles not on either list are regarded as unobserved in that run.

In low-template DNA (or LTDNA) profiling, each epg can be affected by stochastic effects such as dropin, dropout and stutter [1]. To help assess stochastic effects, it is common to perform multiple profiling runs, possibly varying the laboratory conditions but these are nevertheless referred to as replicates. Joint likelihoods for multiple replicates are obtained by assuming that the replicates are independent conditional on the genotypes of all contributors and parameters *ϕ* such as the amounts and degradation levels of DNA from each contributor [2]. We can write

(2) $$\mathrm{Pr}(E|H)=\sum_{j}\mathrm{Pr}(G_{j})\prod_{i}\mathrm{Pr}(R_{i}|G_{j},\phi ),$$

where *R*_*i*_ is the set of allele designations in the *i*th replicate run of the crime scene profile (CSP), $G_{j}$ denotes the *j*th set of contributor genotypes, and the summation is over all possible sets of contributor genotypes under *H*. Pr$\left(G_{j}\right)$ is computed under a standard population genetics model [1]. The unknown parameters *ϕ* can be replaced with estimates, or eliminated by maximisation or integration with respect to a prior distribution.

Currently, there are only limited possibilities to check the validity of an algorithm for evaluating an LTDNA LR (henceforth ltLR). One approach is to evaluate the ltLR when Q is repeatedly replaced by a random profile [3]. In that case *H*_*p*_ is false and we expect the majority of computed ltLRs to be small. Here, we propose to investigate a performance indicator for ltLR algorithms when *H*_*p*_ is true. Under *H*_*d*_, it may occur that $G_{X}=G_{Q}$, where $G_{X}$ and $G_{Q}$ denote the genotypes of X and Q. This occurs with probability *π*_Q_, the match probability for Q. Since Pr$\left(E|H_{d},G_{X}=G_{Q})=\mathrm{Pr}(E|H_{p}\right)$, it follows that [4]

(3) $$\mathrm{ltLR}=\frac{\mathrm{Pr}(E|H_{p})}{\mathrm{Pr}(E|H_{d},G_{X}=G_{Q})\pi_{Q}+\mathrm{Pr}(E|H_{d},G_{X}\neq G_{Q})(1-\pi_{Q})}\leq \frac{1}{\pi_{Q}}.$$

We will refer to 1/*π*_Q_ as the inverse match probability (IMP).

Consider first that Q is the major contributor to an LTDNA profile. Intuitively, if *E* implies that $G_{X}=G_{Q}$ then equality should be achieved in Eq. (3). The key idea of this paper is that if *H*_*p*_ is true then increasing numbers of LTDNA replicates should provide increasing evidence that $G_{X}=G_{Q}$, and so the ltLR should converge to the IMP. This holds even for mixtures if Q is the major contributor, since differential dropout rates should allow the alleles of Q to be identified from multiple replicates. However, any inadequacies in the underlying mathematical model or numerical approximations may become more pronounced with increasing numbers of replicates, preventing the ltLR from approaching the IMP. Therefore we propose to consider convergence of the ltLR towards the IMP as the number of replicates increases as an indicator of the validity of an algorithm to compute the ltLR when Q is the major contributor.

If Q is not the major contributor, even for many replicates there may remain ambiguity about the alleles of Q so that there remains a gap between the ltLR and IMP. However, the bound (3) still holds, and there is a useful guide to the appropriate value of the ltLR provided by the mixture LR for good-quality CSPs computed using only presence/absence of alleles [5]. If under *H*_*p*_ the contributors are Q and U, where U denotes an unknown, unprofiled individual, and *H*_*d*_ corresponds to two unknown contributors X and U, an example of a mixture LR is

(4) $$\mathrm{mixLR}=\frac{\mathrm{Pr}(\mathrm{CSP}=\mathrm{ABC},G_{Q}=\mathrm{AB}|Q,U)}{\mathrm{Pr}(\mathrm{CSP}=\mathrm{ABC},G_{Q}=\mathrm{AB}|X,U)}=\frac{\mathrm{Pr}(G_{U}\;\mathrm{is}\mathrm{one}\mathrm{of}\mathrm{AC},\mathrm{BC},\mathrm{CC})}{\mathrm{Pr}((G_{X},G_{U})\;\mathrm{is}\mathrm{one}\mathrm{of}(\mathrm{AA},\mathrm{BC}),(\mathrm{AC},\mathrm{BB}),(\mathrm{AB},\mathrm{CC}),(\mathrm{AB},\mathrm{AC}),(\mathrm{AB},\mathrm{BC}),(\mathrm{AC},\mathrm{BC}))},$$

where within-pair ordering is ignored in the denominator. Under the standard population genetics model [6], [7] and setting *F*_*ST*_ = 0, the mixLR for this example is

(5) $$\frac{\mathrm{Pr}(\mathrm{CSP}=\mathrm{ABC},G_{Q}=\mathrm{AB}|Q,U)}{\mathrm{Pr}(\mathrm{CSP}=\mathrm{ABC},G_{Q}=\mathrm{AB}|X,U)}=\frac{2p_{A}+2p_{B}+p_{C}}{6p_{A}p_{B}(p_{A}+p_{B}+p_{C})},$$

where the *p* are population allele probabilities. As expected, mixLR < IMP = 1/2*p*_*A*_*p*_*B*_. See Ref. [8] for further details and examples. Note that the mixLR does not use peak height information.

Multiple LTDNA replicates should allow identification of all alleles present in any contributor, and hence the ltLR should reach the mixLR. In fact, ltLR will typically exceed mixLR because the alleles of different contributors may be distinguished over the multiple replicates through differential dropout rates. Indeed, Ref. [9] propose subsampling to generate different mixture ratios in low-template replicates as a strategy to assist mixture deconvolution. We cast light on this possibility below by considering a real CSP that has been profiled using multiple replicates at two different levels of sensitivity. More generally, we examine the behaviour of ltLR in relation to mixLR and IMP, and the utility of each of these for verifying the validity of ltLR computations.

likeLTD is an open-source R package that computes likelihoods for low-template DNA profiles [10]. likeLTD allows for the designation of epg peaks as uncertain in addition to the usual allelic/non-allelic classification, but does not directly use epg peak heights. Uncertain alleles are treated as if they were masked in calculation of the likelihood: the presence/absence of the allele is regarded as unknown. The effect of an uncertain call on calculation of the likelihood is illustrated in Table 1. When B is called as uncertain rather than absent and the hypothesised contributor has a B allele, a dropout term D is removed from the likelihood because the dropout status of B is unknown. We use likeLTD here both to confirm its good performance in computing ltLRs, and to illustrate the value of the IMP as a strict upper bound and the mixLR as an approximate lower bound. We apply likeLTD to lab-based profiling replicates, simulated replicates, and replicates obtained by re-sampling the five actual replicates of a real CSP.

**Table 1:** Likelihood calculations for a CSP when the queried contributor Q has genotype AB and [] indicates an allele designated as uncertain. *L*_*p*_ is the likelihood under the prosecution hypothesis, and *D* is the dropout probability. Under *H*_*d*_ are possible genotypes for the alternative contributor X, where Z is any other allele. *L*_*d*_ is the corresponding contribution to the likelihood under the defence hypothesis, where *p*_*x*_ is the probability of allele x, and *D*_2_ is the homozygote dropout probability.

CSP	*L*_*p*_	*H*_*d*_	*L*_*d*_
A	*D*(1 − *D*)	AA	$p_{A}^{2}(1-D_{2})$
		AZ	2*p*_*A*_(1 − *p*_*A*_)*D*(1 − *D*)

A[B]	1 − *D*	AA	$p_{A}^{2}(1-D_{2})$
		AB	2*p*_*A*_*p*_*B*_(1 − *D*)
		AZ	2*p*_*A*_(1 − *p*_*A*_ − *p*_*B*_)*D*(1 − *D*)

Throughout this paper, ltLR, mixLR and IMP will be reported in units of bans, which is a base 10 logarithmic scale introduced as a measure of weight of evidence by Alan Turing during his wartime code breaking work [11]. Thus 6 bans corresponds to an LR of 1 million on the natural scale.

## 2 Materials and methods

### 2.1 Laboratory replicates

Cheek swab samples were obtained from five volunteers, and DNA was extracted using a PrepFiler Express BTA™ Forensic DNA Extraction Kit and the Life Technologies Automate Express™ Instrument as per the manufacturer's recommendations. The samples were then quantified using the Life Technologies Quantifiler^®^ Human DNA Quantification kit as per the manufacturer's recommendations.

Each sample was serially diluted on a log _10_ scale, and then amplified using the AmpFℓSTR^®^ SGM Plus^®^ PCR kit as per the manufacturer's recommendations on a Veriti^®^ 96-Well Fast Thermal Cycler.

An ABI 3130 Sequencer was used to analyse 1 μL of the PCR products, with 10 second injections at 3 kV; these settings were used for all subsequent analyses. The results returned from the 3130 sequencer were analysed using GeneMapper^®^ ID v3.2 to determine which samples were suitable for further use.

For the one-contributor investigation eight replicates of each of three conditions were created (Table 2). The conditions were created to investigate increasing dropout rate. For the 500 pg and 60 pg conditions, one-contributor hypotheses were compared, B under *H*_*p*_ and X under *H*_*d*_, while for the 15 pg condition dropin was also modelled under both hypotheses (Table 3).

**Table 2:** Sample preparation and genotyping protocol for all conditions examined in the lab-based experiments (described in Table 3). Each condition was replicated eight times. The initial DNA concentration (column 3), dilution (column 4) and volume (column 5) generate approximately the DNA mass indicated in column 6. Columns 7 and 8 show the number of PCR cycles and the volume of PCR product added to each well for the genotyping. Columns 9 and 10 show the ratio of Hi-Di™ formamide to GeneSan™ 400HD ROX™ and the volume of the mixture added to each well. Apmr stands for as per manufacturers recommendations.

Condition	Contributor	Init. conc. (ng μL^−1^)	Dilution (%)	Volume (μL)	Mass (pg)	Cycles	Product (μL)	Formamide: ROX	F/ROX mixture (μL)
(i)	B	31.0	1	1.6	500	28	apmr	apmr	apmr
(ii)	B	31.0	0.1	2.0	60				
(iii)	B	31.0	0.01	5.0	15				

(iv)	A	23.0	1	17.6	500	28	apmr	apmr	apmr
	C	18.1	0.1	16	30				
(v)	A	23.0	0.1	22.4	60	28	apmr	apmr	apmr
	C	18.1	1	22.0	500				

(vi)	A	23.0	0.1	2.7	60	28	apmr	apmr	apmr
	B	31.0	0.1	2.0	60				
	C	18.1	0.1	3.5	60				
(vii)	A	23.0	0.1	2.7	60	28	1	600:1	9
	B	31.0	0.1	2.0	60				
	C	18.1	0.1	3.5	60				
(viii)	A	23.0	0.1	2.7	60	28	9	366:1	11
	B	31.0	0.1	2.0	60				
	C	18.1	0.1	3.5	60				
(ix)	A	23.0	0.1	2.7	60	30	apmr	apmr	apmr
	B	31.0	0.1	2.0	60				
	C	18.1	0.1	3.5	60

**Table 3:** Experimental conditions and hypotheses compared. pg denotes picograms and measures DNA mass; Pr(*D*) denotes the probability of dropout for a heterozygote allele, while Pr(C) denotes the probability of dropin. Pr(unc) indicates the probability of designating a CSP allele as uncertain. *υ* indicates the number of uncertain dropins per locus per replicate; see text for further details of “Condition”. Q denotes the queried contributor, who is one of A, B or C as indicated in parentheses. X is an unknown alternative to Q under *H*_*d*_, while U1 and U2 are unknown contributors under both *H*_*p*_ and *H*_*d*_.

Study	# Contributors	Condition	*H*_*p*_	*H*_*d*_
Lab-based	1	500 pg (i)	Q (B)	X
		60 pg (ii)	Q (B)	X
		15 pg (iii)	Q (B) + dropin	X + dropin
	2	A=500 pg; C=30 pg (iv)	Q (A) + dropin	X + dropin
			Q (A) + U1	X + U1
			Q (C) + U1	X + U1
		A=60 pg; C=500 pg (v)	Q (C) + dropin	X + dropin
			Q (C) + U1	X + U1
			Q (A) + U1	X + U1
	3	28 cycles (vi)	Q (A) + U1 + U2	X + U1 + U2
		Phase 1 (vii)	Q (A) + U1 + U2	X + U1 + U2
		Phase 2 (viii)	Q (A) + U1 + U2	X + U1 + U2
		30 cycles (ix)	Q (A) + U1 + U2	X + U1 + U2

Simulation	1	Pr_*B*_(*D*) = 0; Pr(*C*) = 0	Q (B)	X
		Pr_*B*_(*D*) = 0.4; Pr(*C*) = 0.05	Q (B) + dropin	X + dropin
		Pr_*B*_(*D*) = 0.8; Pr(*C*) = 0.05	Q (B) + dropin	X + dropin
		Pr(unc) = 0.8; *υ*∼ Pois(*λ* = 1)	Q (B)	X
		Pr(unc) = 0.4; *υ*∼ Pois(*λ* = 1)	Q (B)	X
	2	Pr_*A*,*C*_(*D*) = {0.2, 0.8}; Pr(*C*) = 0	Q (A) + dropin	X + dropin
			Q (A) + U1	X + U1
			Q (C) + U1	X + U1
		Pr_*A*,*C*_(*D*) = {0.2, 0.6}; Pr(*C*) = 0	Q (A) + dropin	X + dropin
			Q (A) + U1	X + U1
			Q (C) + U1	X + U1
	3	Pr_*A*,*B*,*C*_(*D*) = {0.8,0.5,0.2}; Pr(*C*) = 0	Q (A) + U1 + U2	X + U1 + U2
		Pr_*A*,*B*,*C*_(*D*) = {0.5,0.5,0.5}; Pr(*C*) = 0	Q (A) + U1 + U2	X + U1 + U2
		Pr_*A*,*B*,*C*_(*D*) = {0.2,0.5,0.8}; Pr(*C*) = 0	Q (A) + U1 + U2	X + U1 + U2

Real-world	≥3	Standard and sensitive	Q + U1 + U2	X + U1 + U2
		Standard only	Q + U1 + U2	X + U1 + U2
		Sensitive only	Q + U1 + U2	X + U1 + U2

For the two-contributor investigation eight replicates of each of two conditions were created (Table 2). The major and minor contributors were reversed between conditions, with an increased DNA contribution from the minor. These samples were amplified and analysed as described previously. Two-contributor hypotheses were compared, with each of A and C in turn playing the role of Q, while the other contributor was treated as unknown. Additionally one-contributor-plus-dropin hypotheses were compared, with only the major contributor playing the role of Q (Table 3).

For the three-contributor investigation eight replicates of each of four conditions were created (Table 2). The conditions were created to investigate different profiling protocols. The Phase 1 and Phase 2 conditions are post-PCR purification protocols designed to enhance the sensitivity of detection of the standard protocol [12], and both involve concentrating the post-PCR product using an Amicon^®^ PCR microcon unit according to the manufacturer's recommendations. Phase 1 enhancement increases the amount of formamide in the mixture compared to the manufacturer's recommendations, while Phase 2 enhancement increases the amount of DNA, formamide and ROX compared to Phase 1. For all four conditions (30 cycles, 28 cycles, Phase 1, and Phase 2), three-contributor hypotheses were compared, with A playing the role of Q and the other contributors treated as unknown (Table 3). Dropin was not modelled under either hypothesis, although dropin was included in the simulations. This reflects a realistic challenge for few replicates with multiple contributors, whereby any dropin alleles may be wrongly attributed to one of the contributors. However the incorrect model will lead to deterioration of inferences for larger numbers of replicates.

### 2.2 Simulated replicates

All of the conditions that we now describe were simulated in eight replicates, with the whole simulation being performed five times. Initially a number of single-contributor CSPs were simulated using the profile of individual B. The first condition investigated was a “perfect match”, in which all eight replicates generated exactly the profile of B. Next, we introduced mild dropout (Pr(*D*) = 0.4) and severe dropout (Pr(*D*) = 0.8) of the alleles of B, in each case with dropins included at rate Pr(*C*) = 0.05 (at most one dropin per locus per replicate). The homozygous dropout probability was set equal to Pr(*D*)^2^/2, as suggested by [13]. We then examined the effect of uncertain allele designations by randomly designating some alleles of B as uncertain, first with Pr(unc) = 0.4 and then Pr(unc) = 0.8. In both conditions, at each locus and in each replicate a Poisson mean one number of alleles not in the profile of B was also designated as uncertain, with types randomly selected according to frequencies in the UK Caucasian database. For all these simulated profiles, one-contributor hypotheses were compared, B under *H*_*p*_ and X under *H*_*d*_.

Next two-contributor CSPs were simulated, based on the profiles of A and C. Two conditions were simulated, both used Pr_*A*_(*D*) = 0.2, while Pr_*C*_(*D*) was initially 0.8 and then 0.6. Dropin was not simulated. For shared alleles the dropout probability was the product of the dropout probabilities for each contributor having that allele. Two-contributor hypotheses were compared, with each of A and C in turn taking the role of Q, while the other was treated as unknown in the analysis. Additionally one-contributor-plus-dropin hypotheses were compared, only for A playing the role of Q (Table 3).

Three-contributor CSPs were then simulated under three conditions, with dropout probabilities for Donors A, B and C as shown in Table 3. Dropin was included as for the one-contributor simulations. Three-contributor hypotheses were compared, with A playing the role of Q and the other two contributors being treated as unknown.

### 2.3 Crime case replicates

We used a CSP from an actual crime investigation, consisting of five replicates: two using standard SGM+ profiling and three generated using an LCN protocol with 34 PCR cycles (Table 4). This example was submitted to us for likeLTD analysis, and as is typical only limited information about the profiling protocol was provided by the profiling lab. These details are not required by likeLTD because it estimates the unknown parameters from the CSP allele designations. We re-sampled the five actual replicates to generate simulated profiles with up to eight replicates, consisting of standard replicates only, sensitive replicates only, or both. Six distinct alleles were observed at locus D8, but no more than three replicated alleles were observed at any locus. Three-contributor hypotheses were compared, with all contributors unknown under *H*_*d*_, and no dropin (Table 3).

**Table 4:** Five replicates of a crime scene profile, three from a sensitive LTDNA profiling technique and two from standard DNA profiling. Alleles shown in [] were called as uncertain.

Locus	Sensitive profiling			Standard profiling	
	Run 1	Run 2	Run 3	Run 4	Run 5
D3	16, [15]	16, [15]	16, 18, [15]	16	16
vWA	15, 16, [17]	15, [14]	15, 18, [14]	15	15
D16	9	9	9, 11, [10]	9	9
D2	17, 19, 24	16, 17, 24,[23]	17, [16]	24	24
D8	8, 13, 15, 16	8, 12, 13, 16, [15]	8, 13, 14, 16, [15]	[8]	
D21	30, 32, 33.2	32, 32.2, 33.2	32, 32.2, 33.2, 34, [31]	[32], [32.2]	[33.2]
D18	12, 17	12, 17, 19	12, 17, [11], [16]	[17]	17
D19	14, 21, [13]	11, 14, [13]	14, [13]	14	14
TH01	6, 9.3	6, 9.3	6, 8, 9.3	[6], [9.3]	[6]
FGA	21	21, [20]	21, 20	21	

## 3 Results

### 3.1 One contributor

#### 3.1.1 Lab-based

**Fig. 1:**
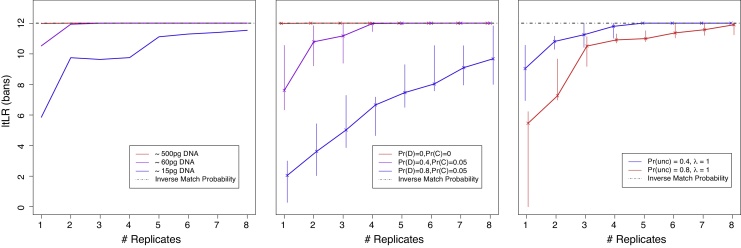
The ltLR shown on a logarithmic scale (in bans) from one-contributor CSPs evaluated using from one up to eight replicates. Left: lab-based replicates, with DNA template (in pg) as shown in the legend box. Middle: simulated replicates with dropout (probability Pr(D)) and dropin (probability Pr(C)); the plotted points represent the median from five repetitions of the simulation, and the vertical bars show the range. Right: simulated replicates with uncertain allele calls (probability Pr(unc) for a true allele to be uncertain, and a Poisson (rate *λ*) number of non-alleles labelled as uncertain at each locus.

For the good-template experiments (500 pg), Fig. 1 (left) shows that the ltLR equals the IMP for all numbers of replicates (one through eight). This is the expected result, and the exercise shows that in this simple setting there is no deterioration in the quality of the computed LR for large numbers of replicates. Low DNA template (60 pg) generates an ltLR about 1.6 bans below the IMP for one replicate, but the gap is very small for two replicates and is negligible for larger numbers of replicates. For very low DNA template (15 pg) the ltLR is just under 6 bans for a single replicate, about 6 bans below the IMP. Replicate profiling substantially narrows the gap, but does not completely close it, with a difference of about 3 decibans remaining at eight replicates.

#### 3.1.2 Simulation

The corresponding simulation studies show broadly similar trends to the lab-based data. For both the perfect match (Pr(*D*) = 0) and mild dropout (Pr(*D*) = 0.4) conditions, the median ltLR rapidly reaches the IMP but does not exceed it, while under severe dropout (Pr(*D*) = 0.8) the median ltLR rises towards the IMP but does not reach it (Fig. 1, middle). For the low and high rates of uncertain calls, the IMP is approximately reached at a five and eight replicates, respectively (Fig. 1, right).

### 3.2 Two contributors

#### 3.2.1 Lab-based

**Fig. 2:**
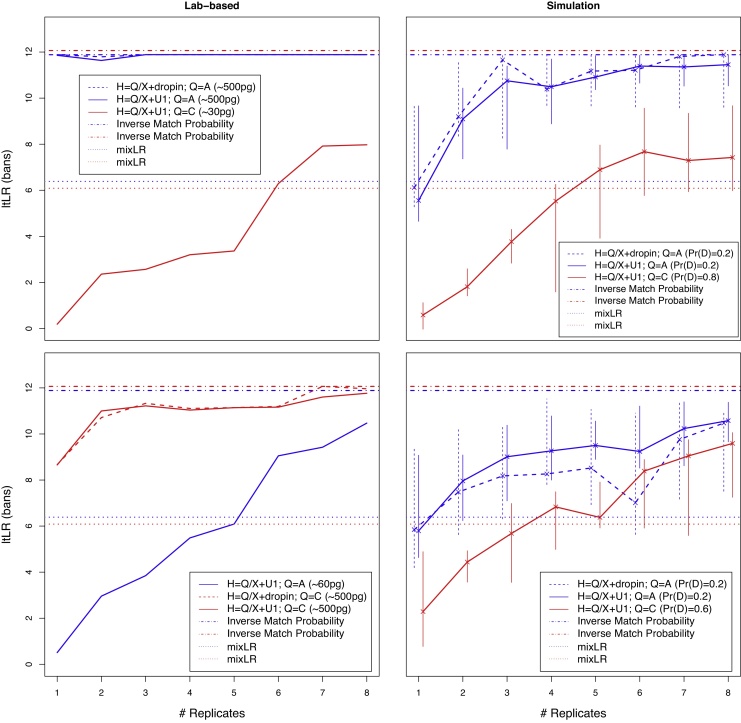
The low-template likelihood ratio (ltLR) from two-contributor CSPs profiled at up to eight replicates. Left: lab-based replicates, with the DNA template from the minor contributor greater in the lower panel (see legend boxes). Right: simulation-based replicates, with the minor contributor having reduced dropout in the lower panel. The simulated CSPs were generated from the profiles of Donors A and C, and the line colours on the graph indicate whether the queried individual (Q) is A (blue) or C (red). Solid lines indicate a two-contributor analysis, with the non-Q individual regarded as unknown (U1). Dashed lines indicate a one-contributor analysis that also allows for dropin (only for Q the major contributor). The inverse match probability is shown with dot-dash lines, coloured according to Q. The mixLR is shown with dotted lines, coloured according to Q. In the legend boxes, H indicates the hypotheses with X an unknown alternative to Q, and Pr(D) indicates the probability of dropout.

When the minor contributor provides only 30 pg of DNA (Fig. 2, top left panel), then if Q is the major contributor the ltLR is very close to the IMP for all numbers of replicates, whereas if Q is the minor contributor then there remains a substantial gap between ltLR and IMP even at eight replicates. However, even with this very low template, the ltLR exceeds the mixLR beyond five replicates. When the major and minor contributors are reversed, and the amount of DNA from the minor is doubled (Fig. 2, bottom left), then if Q is the minor contributor the ltLR substantially exceeds mixLR from six replicates and rises to within two bans of the IMP at eight replicates. Under both conditions, the two-contributor analysis gives a very similar result to the one-contributor-with-dropin analysis.

#### 3.2.2 Simulation

When the minor contributor is subject to high dropout (Fig. 2, top right), then if Q is the major contributor the ltLR exceeds the mixLR after one replicate, and rises rapidly to within about 2 bans of the IMP, but the gap narrows only slowly thereafter. The one-contributor-plus-dropin analysis gives an ltLR that is broadly similar to the two contributor analysis, but with a wider range indicating greater variability. If Q is the minor contributor, the median ltLR increases rapidly from a low base, and appears to stabilise after about five replicates, about four bans below the IMP but exceeding the mixLR. The range increases after three replicates, and remains high up to eight replicates.

With reduced dropout for the minor contributor (Fig. 2, bottom right), inferring the presence of a major contributor Q is harder because of additional masking by the minor contributor. The median ltLR in both the two contributor and one-contributor-plus-dropin analyses eventually reaches within 2 bans of the IMP, with the latter showing a greater range. Conversely, the lower dropout rate leads to improved inference for a minor contributor Q, with the median ltLR rising to about three bans below the IMP at eight replicates, and exceeding the mixLR from four replicates. Interestingly, after six replicates the range of the minor contributor ltLR overlaps the range for the major contributor.

### 3.3 Three contributors

#### 3.3.1 Lab-based

**Fig. 3:**
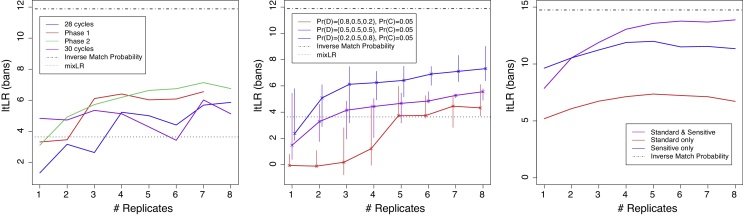
The low-template likelihood ratio (ltLR) for three-contributor crime stains profiled with one to eight replicates. Left: laboratory replicates using four lab techniques indicated in the legend box and described further in Section 2. Middle: simulated replicates with dropout rates for the three contributors as shown in the legend box against Pr(D), the first value being for the queried contributor. Pr(C) is the dropin probability. Right: re-sampled actual crime-stain replicates; the original data are two standard profiling replicates, and three replicates using enhanced sensitivity. The ltLR returned from a perfect replicate of the contributors (consisting of every allele from each contributor) is shown with dotted lines; this is not possible for the real-world case, as the true contributors are unknown.

The 30 PCR cycles condition gives the highest ltLR at one replicate but little improvement with additional replicates (Fig. 3, left). The other amplification methods do show an increasing ltLR trend with additional replicates, but in no case did the ltLR reach within four bans of the IMP. As expected, the ltLR for both phase 1 and phase 2 enhancement exceeds that for standard 28 PCR cycles at all numbers of replicates, and phase 2 enhancement ltLR typically gives a small improvement over phase 1 enhancement. For 30 PCR cycles, the ltLR exceeds the mixLR for a single replicate but dips slightly below it at six replicates. For the other conditions, the mixLR is always exceeded from four replicates.

#### 3.3.2 Simulation

All three curves in Fig. 3 (middle) show an increasing trend with number of replicates, with the median ltLR being in the expected order throughout (decreasing ltLR with increasing dropout for Q). The median ltLR exceeds the mixLR after one replicate (low dropout), after two replicates (medium dropout) and after four replicates (high dropout). The range is often wide, reflecting a strong dependence of the ltLR on the details of the simulation (in particular the number of alleles shared across contributors).

#### 3.3.3 Real-world case

The ltLR returned when only standard or only sensitive replicates are used shows a similar trend, but nearly five bans lower for the standard replicates (Fig. 3, right). For three or more replicates, using mixed types of replicates is superior even to only using sensitive replicates, coming to within two bans of the IMP. This partly reflects the limited pool of replicates used in the actual crime case, but suggests that using different sensitivities in the profiling replicates may convey an advantage due to different contributors being better distinguished.

## 4 Discussion

We have shown that ltLR computed by likeLTD is bounded above by the IMP in every condition considered, as predicted by theory (Eq. (3)). That the bound is often tight when Q is the major contributor (Fig. 1, Fig. 2 (top)) supports the validity of the underlying mathematical model, and its correct implementation in the likeLTD software. Our results should help counter any misconception that combining multiple noisy profiling replicates only compounds the noise: in fact, multiple noisy replicates can fully recover the genotype of a contributor [14].

A novel feature of likeLTD, is that it can accommodate uncertain allele designations, which diminishes the problem of an all-or-nothing allele call, therefore mitigating the problem highlighted by [15] of choosing a detection threshold. We have shown (Fig. 1 (right)) that introducing many uncertain allele calls leads to ltLRs that satisfy the bound, which is reasonably tight with as few as three replicates even when 80% of true alleles are designated as uncertain and there are also multiple uncertain non-alleles.

We have further shown that mixLR, the LR computed from knowing every allele that is represented in the profile of at least one contributor to the CSP, is often surpassed after only a handful of replicates. Then, multiple LTDNA replicates provide stronger evidence than a single good quality profile correctly representing the alleles of all contributors, which occurs because the alleles of different contributors can to some extent be distinguished through differential dropout rates in multiple replicates. These results lend support in principle to the proposal of [9].

Fig. 2 shows that, for two-person mixtures, the analysis assuming one-contributor-plus-dropin gave a very good approximation for the lab-based replicates (left panels), and a reasonably good approximation for the simulation replicates, but with more variable ltLR values, as indicated by the wider range.

### 4.1 Choice of profiling technique

We generated three-contributor CSPs in order to compare different LTDNA profiling techniques. We chose the most challenging condition in which all three contribute the same DNA template, making it impossible to deconvolve the mixture into the genotypes of individual contributors. We found that PCR performed with 28 cycles (regardless of enhancement) is preferable to 30 cycle PCR beyond one replicate (Fig. 3). More PCR cycles introduces more stochasticity in the results, as stated in the AmpFℓSTR^®^ SGM Plus^®^ PCR Amplification Kit user guide. We found that enhancement of the post-PCR sample is advantageous, with Phase 2 enhancement providing a small further improvement over Phase 1 (Fig. 3). These results support those of Forster et al. [16], who demonstrated that increasing PCR cycles increases the size of stutter peaks and the incidence of dropin; we observed no improvement in the WoE for 30 PCR cycles, possibly due to these stochastic effects.

The results from the real crime case (Fig. 3, right) suggest that if possible, a mixture of LTDNA replicates with differing sensitivities should be employed, as this allows better discrimination between the alleles of different contributors and hence a higher ltLR than the same number of replicates all using the same sensitivity.

### 4.2 Use of replicates

Splitting the sample reduces the quality of results expected in each replicate compared with that which would be obtained from a single profiling run using all available DNA. Grisedale and van Daal [17] favour use of a single run, but their comparison was with a consensus sequence obtained from multiple replicates, rather than the more efficient statistical analysis available through analysing individual replicates. Our results show increasing information obtained from additional replicates, which may tilt the argument towards use of multiple replicates but we have not done a comparison directly addressing this question. To fully test the performance of likeLTD in relation to mixLR and IMP we have used up to eight replicates. Taberlet et al. [18] suggest seven replicates to generate a quality profile when the amount of DNA is low, but this many replicates is rarely available for low-template crime samples [15].
